# Cost-Effectiveness of Prostate Cancer Detection in Biopsy-Naïve Men: Ultrasound Shear Wave Elastography vs. Multiparametric Diagnostic Magnetic Resonance Imaging

**DOI:** 10.3390/healthcare10020254

**Published:** 2022-01-28

**Authors:** Mehdi Shiva, Cheng Wei, Hassan Molana, Ghulam Nabi

**Affiliations:** 1 Blavatnik School of Government, University of Oxford, Oxford OX2 6GG, UK; 2 Division of Imaging Science and Technology, School of Medicine, University of Dundee, Ninewells Hospital, Dundee DD1 9SY, UK; c.wei@dundee.ac.uk (C.W.); g.nabi@dundee.ac.uk (G.N.); 3 School of Social Science, University of Dundee, Dundee DD1 4NH, UK; h.h.molana@dundee.ac.uk

**Keywords:** cost-effectiveness analysis, shear wave elastography, magnetic resonance imaging, prostate cancer, quality-adjusted life years

## Abstract

This exploratory study investigates the cost-effectiveness of ultrasound shear wave elastography (SWE) imaging in comparison to pre-biopsy multiparametric magnetic resonance imaging (mpMRI) in men with suspected prostate cancer. This research is motivated by the early evidence of the good performance of SWE in distinguishing cancerous from benign prostate tissues. We used a decision analysis model representing the care-pathways of men referred with a high prostate specific antigen (PSA) and/or abnormal digital rectal examination (DRE) in a UK setting from the payer’s perspective with results reported in 2016 GBP. We then appraised the cost-effectiveness of a novel approach based on SWE compared to the more conventional and widely practiced mpMRI-based approaches using data reported in the literature. Deterministic and probabilistic sensitivity analyses were used to address uncertainty regarding the parameter values utilised. Our exploratory results implied that SWE approach yielded an additional quality-adjusted life year (QALY) at the cost of GBP 10,048 compared to the standard mpMRI-based approach in the UK. This is lower than the official willingness to pay threshold of GBP 20,000 (the UK healthcare system guidelines) and is therefore a suitable replacement for the current practice. Sensitivity analyses confirmed the robustness of our results.

## 1. Introduction

In many countries across the world, especially those with advanced healthcare systems, cost-effectiveness analyses form the backbone of decision making on the allocation of limited resources to maximise efficiency and health benefits. Ideally, these analyses are revised when a serious alternative method of diagnosis and/or treatment of a considerable disease is developed. Pre-biopsy imaging procedures are desirable in prostate cancer treatment as biopsy is an invasive procedure with many possible side effects, e.g., acute bleeding, sepsis, urinary retention and even death [1]. A combination of the high prevalence of prostate cancer and the poor diagnostic accuracy of the prostate-specific antigen (PSA) underscores the importance of diagnosis methods and calls out for further attempts to improve (reduce) their performance (cost).

This paper considers the case of prostate cancer where multiparametric magnetic resonance imaging (mpMRI) has an established detection record [2,3] and is recommended by the National Institute for Health and Care Excellence (NICE) as the first-line investigation for people with suspected clinically localised prostate cancer followed up by mpMRI-guided transrectal ultrasound (MRI-TRUS) fusion biopsy when required [4]. However, emerging evidence place shear wave elastography (SWE) as a possible contender [5,6,7]. In two recent lab studies [5,6], using a 3D mould-based approach and radical prostatectomy histology as reference standard, it is shown that SWE accurately detects cancer foci and shows significant differences between cancerous and benign tissue. The proposed SWE-based technique in these studies specially demonstrates a solid performance in picking up anterior tumours in the prostate gland and proves as a reliable approach in estimating the aggressiveness, even outperforming mpMRI [5]. Similar to mpMRI, SWE showed considerably better results in detecting clinically significant cancers and consistently showed a progressive increase in tissue stiffness with grade and Gleason score of disease. In addition to standard functions, SWE also utilises the quantification of stiffness based on colour-coded images on screen, along with conventional ultrasound with much higher resolution (frame rate of 20–40,000/s). This is a much-needed improvement to cover inter-observer variation between radiologists and protocol variations in centres using MRI-based approaches [8].

SWE is also expected to cost less per unit compared to the mpMRI approach as imaging, reporting, and staff training is straightforward and the quantification of SWE images is semi-automatic. This is in opposed to many complexities with the mpMRI approach, namely variations in protocols, dependency on large infrastructural investment, shortage of experienced staff, and quality assurance requirements [9]. There is, however, no evidence to date comparing life-time costs of SWE and mpMRI approaches.

The mpMRI holds a strong evidence-based support in the literature as it is believed to improve prostate cancer detection and reduce unnecessary biopsies by a quarter [10]. There are many studies assessing the cost-effectiveness of mpMRI-based approaches using different pathway scenarios. A recent study [11] described a cost-effectiveness analysis based on the PROMIS study [10] and concluded that practicing mpMRI before biopsy can provide a more accurate diagnosis for men with suspected prostate cancer. This study also suggests that mpMRI is significantly better at identifying clinically significant cancers when compared to transrectal ultrasound (TRUS). However, the PROMIS trial excluded some of the blended methods, such as MRI transrectal ultrasound (MRI-TRUS) fusion biopsy as a comparator in their study. MRI-TRUS fusion biopsy exploits the high accuracy of mpMRI with the real-time capability and ease of TRUS biopsy. It is also considered less expensive than an MRI-guided biopsy (MRGB) and can be performed in a shorter time [12]. This is a major shortcoming given that MRI-TRUS fusion biopsy following mpMRI is a strategy endorsed by the latest NICE guideline [4] and suggested to be cost effective in comparison to other existing strategies [13,14,15,16]. Therefore, the PROMIS results do not encompass all the relevant case scenarios for day-to-day clinical practice.

A number of studies have addressed the question of the cost-effectiveness of mpMRI in prostate cancer detection [14,17,18]. However, to our knowledge, no study to date has compared the cost-effectiveness of mpMRI-based techniques with the SWE approach. In this paper, we follow the CHEERS guidelines [19] to report the economic evaluation of the SWE strategy compared to the two common diagnostic methods which are both based on mpMRI. The choice of comparator is in line with clinical practice in the UK as well as the academic literature supporting it as the most cost-effective method for diagnosing prostate cancer (according to NICE [4], mpMRI is expected to stay as the default procedure at least until 2023–2024). More precisely, we evaluated and compared the cost-effectiveness of the following diagnosis strategies: (i) using mpMRI examination followed by MRI-TRUS fusion biopsy only if the diagnostic MRI result is positive—otherwise no biopsy at all (the current reference practice); (ii) using mpMRI examination followed by MRI-TRUS fusion biopsy if results are positive, standard biopsies (TRUS guided biopsy) otherwise (former most common practice); and (iii) using SWE followed by TRUS guide biopsy in SWE-detected lesions. All three strategies were assumed to be offered to biopsy-naïve men presenting with clinical suspicion of cancer with increased serum PSA and/or abnormal digital rectal examination (DRE). It should be noted that this is an exploratory study, meaning that our findings are at best a starting point for planning more robust, prospective and protocol-driven research to address the key questions outlined in this section.

## 2. Materials and Methods

### 2.1. Model Development

**Figure 1:**
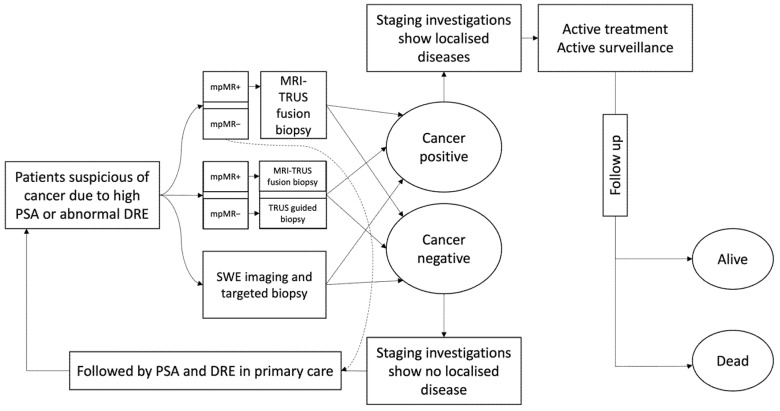
The tree diagram.

We constructed a decision tree to portray the case of a biopsy-naïve patient in whom a prostate biopsy was indicated based on either elevated PSA levels and/or abnormal DRE findings. The process starts when we observe either an increase in PSA or an abnormal DRE in men with lower urinary tract symptoms or during screening. We then proceeded to assess which of the three main diagnosis strategies stated above are most suitable for the NHS, based on using either mpMRI or SWE, which constitute the initial branches of the tree diagram in Figure 1. Given the outcome, each branch then bifurcates into two representative cases where prostate cancer truly exists (tumour +) or does not exist (tumour −). We then used the data to calculate the probabilities and costs of each scenario at the corresponding node. The tree continues to branch out further to cover the biopsy tests that follow the initial MRI or SWE diagnostic examinations and it ends with terminal nodes representing different treatment methods, namely radical prostatectomy (RP), radiotherapy (RT), hormone therapy (HT), a mixture of radio and hormone therapy (RT + HT), and watchful waiting/active surveillance (WW/AS). The sensitivity and specificity of SWE and mpMRI were derived from lab data based on a sample of 12 patients with clinically localised prostate cancer [5]. Each participant had a pre-biopsy mpMRI and SWE imaging and subsequently underwent RP to estimate their diagnostic accuracy. More precisely, the authors of the mentioned lab study adopted a prostate region-wise based analysis—where prostate is divided into twelve regions by imaginary lines dividing each lobe into base, mid and apex; each lobe is then further divided into medial and lateral with respect to the line placed in the middle, effectively creating six regions in each lobe—and the outcome of the corresponding whole-mount RP histopathology (where a whole prostate gland is removed during radical surgery and is then processed by histopathology) for each region was used to label it as cancer (or tumour) negative or positive accordingly. SWE statistics are further confirmed in a larger study on 212 patients [6]. Effectively, the diagnosis and imaging procedures for both insignificant and significant cancers are similar, if not identical; the choice of final treatment differs as insignificant cancers usually receive milder treatments, such as radiotherapy or watchful waiting/active surveillance.

The decision tree approach for estimating the expected economic cost of each strategy is briefly described below.

Strategy 1: Based on NICE guideline [4], this is the ‘reference practice’ or ‘benchmark strategy’ where an elevated serum PSA and/or abnormal digital examination is followed by mpMRI examination. When a tumour-suspicious area is identified on mpMRI, defined as any score 3 or above based on the Prostate Imaging Reporting and Data System (PIRADS [20]), the patient is scheduled for an MRI-TRUS fusion biopsy. In the case of a negative mpMRI outcome, the patient’s urologists periodically (6–12 monthly) follow up with PSA testing. No standard biopsies are offered in mpMRI negative patients. When MRI-TRUS fusion biopsy determines a tumour, the patient undergoes one of the following treatment methods: RP, RT, HT, a combination of both RT and HT, or WW/AS. The various probabilities of treatment options in men with localised prostate cancer on imaging are listed in Table 1. We did not use MR guided biopsy (MRGB) in any of the strategies as it is not offered clinically on a routine basis in the UK.

Strategy 2: Similar to Strategy 1, an elevated serum PSA and/or abnormal DRE was followed by mpMRI examination. When a tumour-suspicious area is identified on mpMRI, the patient is scheduled for an MRI-TRUS fusion biopsy. However, unlike Strategy 1, TRUS biopsy were offered in MRI negative patients. The rest of the process is identical to described in Strategy 1. This strategy used to be the common practice, but it lost its place to strategy 1 due to recent evidence emerging from cost-effectiveness studies [17].

Strategy 3: This is our ‘experimental’ or ‘index’ strategy where an elevated serum PSA and/or an abnormal DRE is followed by SWE-guided biopsy (this is a TRUS-based SWE-guided biopsy with much higher precision compared to TRUS biopsy—see more in [5,6]). In this strategy, a negative SWE result in the patient is followed up by annual PSA testing with DRE. When a tumour is detected, the patient undergoes the same treatment options as in Strategy 1 (either one of RP, RT, HT, RT + HT, or WW/AS).

We did not use the TRUS biopsy strategy without pre-biopsy mpMRI as this approach is less clinically practiced and is not cost-effective as previously reported [21]. In all strategies, we assumed that patients with a false-negative (FN) test result would have their tumour detected and treated at some point in the future [18]. This assumption was made on the grounds that the patient’s urologist would follow up with PSA testing on a 6–12 monthly basis. Therefore, it is sensible to expect that the health condition of the patient will deteriorate at some point in the future and there will be repeated referrals to the system and subsequent repeated imaging and biopsies and consequently a correct diagnosis. Tumours smaller than 5 mm are more likely to be missed in the initial rounds of diagnosis.

### 2.2. Costs, Probabilities, and QALYs

We obtained the cost data from existing studies and NICE guidelines. The cost data are associated with the following procedures used in this study: (i) a diagnostic MRI examination (mpMRI), (ii) an MRI-TRUS fusion biopsy; (iii) a systematic transrectal ultrasound guided biopsy (TRUS guided biopsy); and (iv) a diagnostic SWE examination (transrectal ultrasound SWE) along with a SWE-guided prostate biopsy. The cost figures are based on per person expenditure that is applicable in the United Kingdom and are mainly derived from NICE publications [4]. The cost values of both mpMRI and SWE-based approaches are inclusive of the amount of time that each specialist spent, as well as the equipment and consumable costs per person (see Table 1). The total lifetime costs of treatment procedures were taken from the literature [21], where the full expected patient-level costs were estimated using a life-time modified Markov model based on data from the literature. It is worth noting that the life-time cost figures are from a study based on a USA setting (the base case being a 65-year-old man) and capture the full expected lifetime costs and benefits that may be a consequence of over- or under-diagnosis and over- or under-treatment. Within the resources available for this early analysis, we were not able to model lifetime costs and outcomes specific to the UK setting. Although not ideal, the U.S. data for lifetime costs and outcomes was available in the format required and is acceptable for use in an incremental analysis where we are interested in relative rather than absolute costs and outcomes (to ensure robustness we used probabilistic sensitivity analysis with a wide range). In addition to this cost data, we also used reported probability data, which are also displayed in Table 1, to construct the respective costs of each diagnostic strategy. All cost estimates were adjusted for UK inflation and were measured in 2016 GBP (data on inflation are from the UK Office for National Statistics and the annual average USD/GBP exchange rate was used to convert the values).

## 3. Results

### 3.1. Costs-Effectiveness Analysis

**Figure A1:**
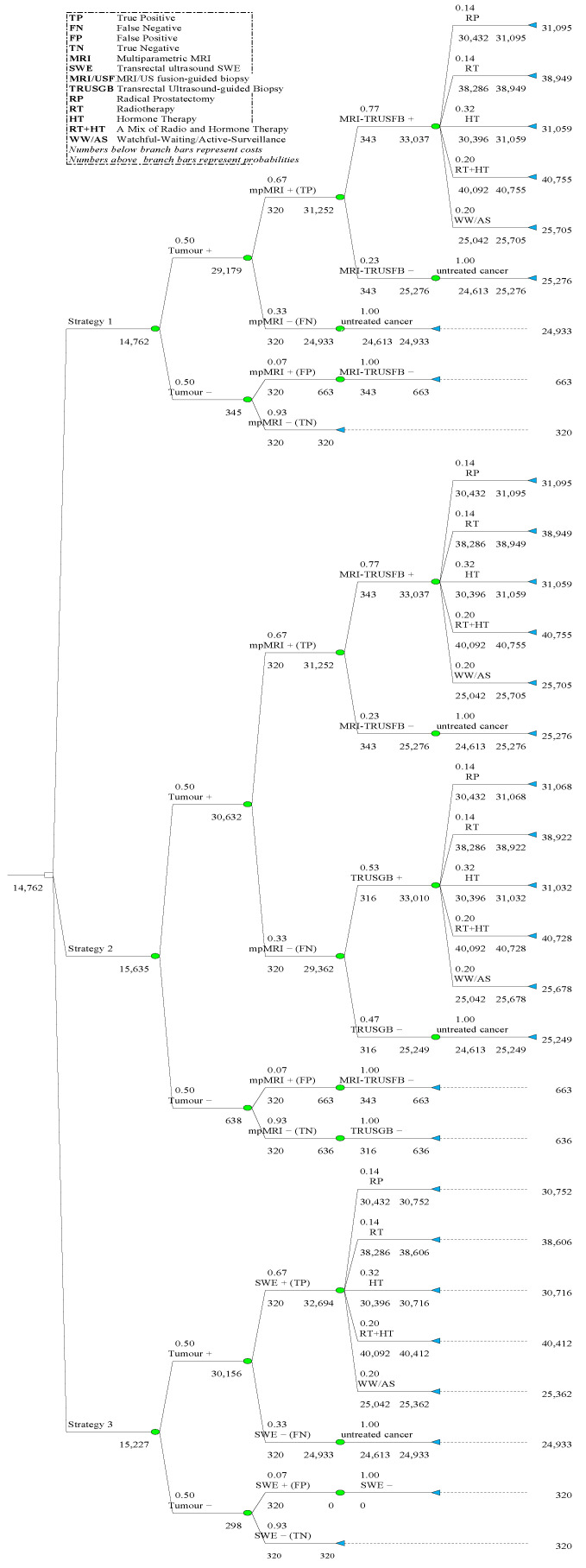
Decision tree of costs.

**Figure A2:**
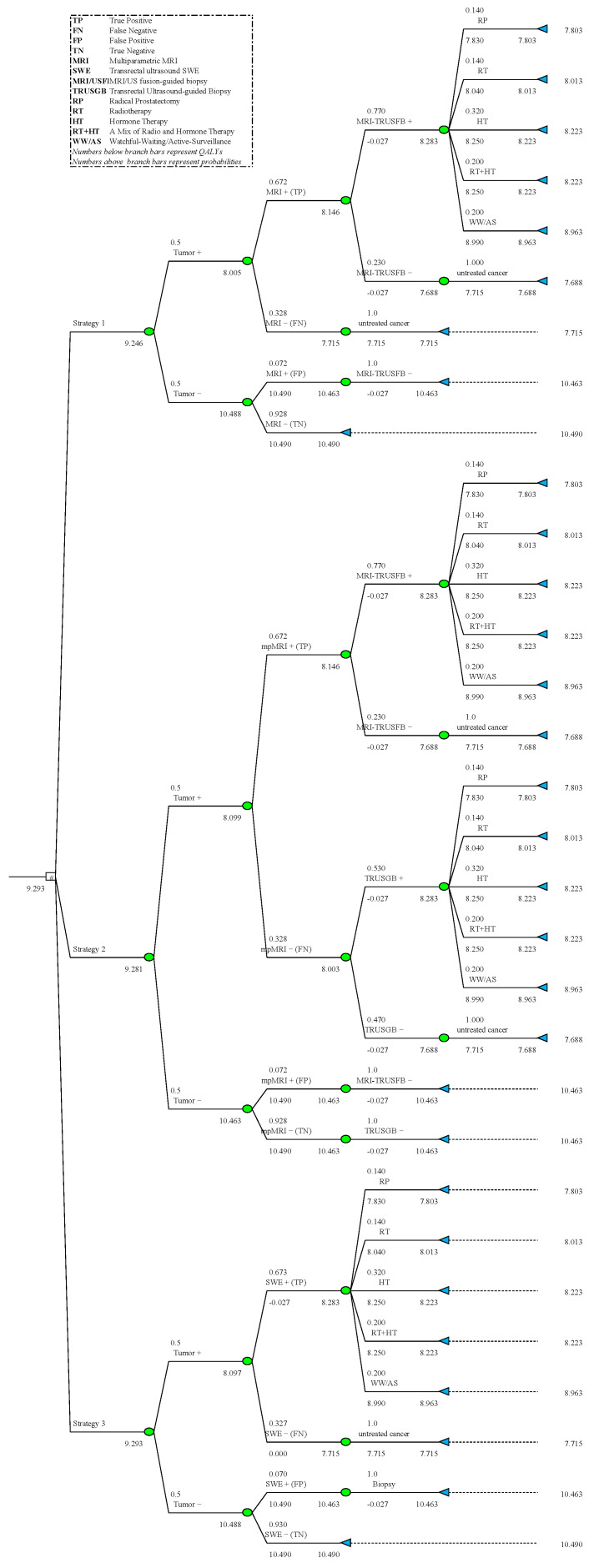
Decision tree of QALYs.

We used quality-adjusted life years (QALY) data from the literature to build the effectiveness side of outcomes in our decision analysis (Table 1). The incremental cost-effectiveness ratios (ICER) were then compared with the UK willingness to pay (WTP) lower threshold of GBP 20,000/QALY, which constitutes the primary outcome measure [23]. The future values of both costs and QALYs were adjusted using an annual discount rate of 3%. The decision trees, which were constructed using TreePlan Software^©^ on the basis of the data reported in Table 1, yielding estimates of the overall costs and QALYs values of the three different strategies, are shown in Figure A1 and Figure A2 in the Appendix A.

Finally, in Table 2 below, we present the incremental values of costs and QALYs, the corresponding lifetime ICERs and two alternative measures of net health benefits (NHB) for the three strategies. According to our model, SWE approach is more effective than the benchmark mpMRI approach, having an incremental effect of 0.046 QALY. However, the benchmark mpMRI approach is less expensive: the average cost per patient is GBP 14,762, whereas the average costs of SWE approach per patient is GBP 15,227, which results in an incremental cost of GBP 465 for SWE approach. As a result, the ICER for SWE approach is GBP 10,048 per QALY gained, which is substantially below the UK official WTP threshold of GBP 20,000 that is exceeded for Strategy 2 with GBP 25,126. Additionally, using the NHB values as a guide, on the whole Strategy 3, which is based on SWE, dominates the other two MRI-based strategies. Overall, relative to Strategy 1, Strategy 3 provides an unambiguously better result.

### 3.2. Sensitivity Analysis

**Figure 2:**
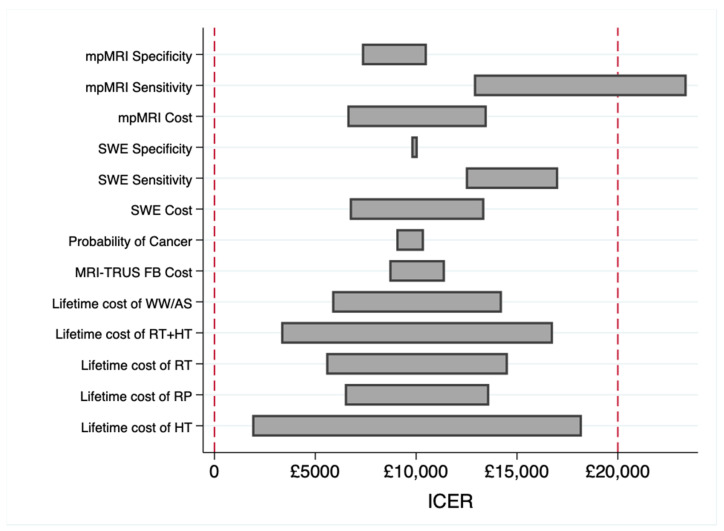
Deterministic sensitivity analysis (tornado diagram).

As Table 2 shows, the results are robust to increasing the WTP threshold for per QALY gain; the SWE-based diagnostic approach (Strategy 3) yielded a higher NHB at both the GBP 20,000 and GBP 30,000 WTP thresholds. In addition, our deterministic sensitivity analysis for all cost and probability factors established the dominance of Strategy 3 in all cases at a GBP 20,000 WTP threshold. The results are shown in Figure 2 below, which were obtained by varying the values of cost or probability in question within an interval [0.5*x*, 1.5*x*], where *x* is the value cost or probability used in the benchmark analysis reported, while keeping other variables constant. It appears that ICER is most sensitive to the sensitivity values of mpMRI and SWE. This indicates that extra attention should be given to sensitivity values of mpMRI and SWE in future cost-effectiveness studies using larger lab datasets. Finally, we have carried out a probabilistic sensitivity analysis on costs, QALYs, and the probability of different treatment methods to obtain confidence intervals for the benchmark values reported in Table 2 (presented inside brackets). In each case, the 95% confidence interval covers the benchmark value, supporting the robustness of our results to variations in costs, QALYs and probabilities used as ingredients.

## 4. Discussion

In this paper, we analysed the cost-effectiveness of an emerging ultrasound technology—the SWE-based tissue stiffness measurement diagnostic method—in comparison to the already established, more conventional, mpMRI-based pathways augmented with the MRI-TRUS fusion biopsy methods for suspected area. To our knowledge, this is the first study to evaluate the cost-effectiveness of different imaging approaches, which includes SWE, for diagnosing prostate cancer in biopsy naïve men. Our early results showed that the SWE-based approach outperformed its more conventional rival. This echoes the NICE report [4] that undermines the cost-effectiveness of contrast-enhanced MR imaging and MR imaging-guided biopsy in detecting prostate cancer.

It is important to note that our study is focused on the SWE-biopsy approach without systematic sampling. This is founded on the belief that SWE could generate a very high negative predictive value could be achieved for clinically significant cancers. It is also worthwhile stressing that cost-effectiveness analysis relies on the WTP thresholds, which tend to vary considerably across different healthcare systems. With an increasing number of technologies and limited resources in centrally funded healthcare systems, such as the NHS in the UK, clinical and cost-effectiveness comparisons play an important role in determining the so called ‘value for money’ associated with each method. The UK Department of Health and NICE guidelines suggest a WTP range of GBP 20,000–30,000 (EUR 22,000–34,000; USD 30,000–45,000) per QALY [24,25]. We take these recommended figures as given and use them in our analysis; whilst they clearly affect our findings, the way these recommended figures are determined lies beyond the scope of our study.

The present study has a few shortcomings that should be addressed in future research. Firstly, we did not distinguish prostate cancers with respect to their significance since the only available data for SWE approach does not allow such disaggregation [5]. The mpMRI sensitivity and specificity was taken from the same lab study to keep consistent with the SWE population and setting. Given the limited body of evidence in the literature on SWE’s performance and costs, we believe that relying on this lab study, which is peer reviewed, is justified for our exploratory study. Future studies might have access to larger datasets with better segregation for the significancy level of tumours. Additionally, our lifetime costs are collected from a U.S. study and this might differ in the UK population. We tried avoiding comparing absolute figures and focused on relative comparisons, but utilising UK figures would strengthen the results. Lastly, as any other exploratory study, the set of assumptions we took might have influenced the results. In the light of these issues, confirmation of which method offers better overall detection and more cost-effective performance requires prospectively collected data with robust reference standards. Therefore, our current work is at best a starting point for planning more robust, prospective and protocol-driven research to address the key questions outlined in this paper.

## 5. Conclusions

Pre-biopsy imaging in men suspected of having prostate cancer in primary care has important clinical benefits. Taking the limitations into consideration, we conclude that SWE is more cost effective and yields added net health benefits when compared to the mpMRI in a UK healthcare setting. Future research is needed to determine whether larger and more comprehensive sets of data on SWE can hold similar cost-effectiveness results.

## Figures and Tables

**Table 1:** Costs, probabilities, and QALYs used in decision analysis.

Parameter	Cost	Source	Probability	Source	QALY	Source
Diagnosis and biopsy						
mpMRI	GBP 320	[4]				
*sensitivity*			0.67	[6]		
*specificity*			0.92	[6]		
SWE	GBP 320	AE ^i^			−0.027 ^ii^	[21]
*sensitivity*			0.67	[6]		
*specificity*			0.93	[6]		
MRI-TRUS fusion biopsy	GBP 343 ^iii^	[4]			−0.027 ^ii^	[21]
*sensitivity*			0.77	[15]		
*specificity*			1.00 ^iv^	[15,21]		
TRUS guided biopsy	GBP 316	[4]			−0.027 ^ii^	[21]
*sensitivity*			0.53	[15]		
*specificity*			1.00 ^iv^	[15,21]		
Treatment						
Radical prostatectomy (RP)	GBP 30,432	[21]	0.14	[22]	7.83	[21]
Radiotherapy (RT)	GBP 38,286	[21]	0.14	[22]	8.04	[21]
Hormone therapy (HT)	GBP 30,396	[21]	0.32	[22]	8.25	[21]
Radiotherapy + hormone therapy (RT + HT)	GBP 40,092	AE ^v^	0.20	[22]	8.25	AE ^vi^
Watchful waiting/active surveillance (WW/AS)	GBP 25,042	[21]	0.20	[22]	8.99	[21]
Undiagnosed, untreated, or later-diagnosed cancer	GBP 24,613	[21]			7.71	[21]
No prostate cancer			0.50	[21]	10.49	[21]

^i^ Authors’ estimate. The transrectal ultrasound SWE unit cost was estimated by the authors and breaks down into cost of current ultrasound examination of prostate plus per patient cost of acquiring the SWE machine. ^ii^ There is a penalty associated with any biopsy procedure. For SWE, this is only applicable when SWE imaging shows tumour positive and therefore a biopsy is initiated. ^iii^ The MRI-TRUS fusion biopsy unit cost breaks down into: TRUSGB unit cost + GBP 27 additional cost of using fusion image registration [4]. ^iv^ This is on the grounds that “*While this is almost certainly an overestimate, its influence on the cost-effectiveness results should not overstated as it is incremental differences between strategies that drive cost-effectiveness results and there is little reason to suspect significant specificity difference between the strategies*” [6]. We have tested the robustness of results against replacing this value by the interval [0.5, 1) in the sensitivity analysis. ^v^ Authors’ estimate. The lifetime cost of RT+HT is estimated by adding the two-year cost of HT (GBP 903 per year) to RT lifetime cost on the grounds that HT is only prescribed in the first two years of this treatment method. ^vi^ Authors’ estimate.

**Table 2:** Cost-effectiveness analysis ^1^.

Strategy	Total QALYs	Incremental ^2^ QALYs	Total Cost	Incremental ^2^ Cost	ICER ^2^	NHB ^3^ (GBP 20k)	NHB ^3^ (GBP 30k)
1	9.246	-	GBP 14,762	-	-	8.5082	8.7542
	[9.09, 9.40] ^4^		[14,103, 15,412] ^5^				
2	9.281	0.0347	GBP 15,635	GBP 873	GBP 25,126	8.4993	8.7599
	[9.04, 9.53] ^4^	[−0.25, 0.107] ^4^	[14,695, 16,450] ^5^	[542, 1203] ^5^	[14,619, 38,653] ^5^		
3	9.293	0.0462	GBP 15,227	GBP 465	GBP 10,048	8.5312	8.7850
	[8.90, 9.68] ^4^	[−0.027, 0.118] ^4^	[13,568, 15,509] ^5^	[195, 733] ^5^	[4075, 16,905] ^5^		

^1^ The values in this table are calculated in double precision by Matlab software (codes are available upon request). ^2^ ICER = cost increment per unit of QALY increment; increments are calculated relative to Strategy 1. ^3^ Net Health Benefit = (QALY − Cost/WTP), based on WTP thresholds of GBP 20,000/QALY and GBP 30,000/QALY. ^4^ 95% confidence interval based on varying probabilities within ±50% range and QALYs within ±20%. ^5^ 95% confidence interval based on varying probabilities within ±50% range and Costs within ±20%.

## Data Availability

Data, material, and codes: Available upon request.
